# Alternariol 9-*O*-methyl ether dimethyl sulfoxide monosolvate

**DOI:** 10.1107/S1600536813012294

**Published:** 2013-05-11

**Authors:** Sreekanth Dasari, Kristin I. Miller, John A. Kalaitzis, Mohan Bhadbhade, Brett A. Neilan

**Affiliations:** a School of Biotechnology and Biomolecular Sciences, University of New South Wales, Sydney, NSW 2052, Australia; b Mark Wainwright Analytical Centre, University of New South Wales, Sydney, NSW 2052, Australia

## Abstract

The title compound (systematic name: 3,7-dihy­droxy-9-meth­oxy-1-methyl-6*H*-benzo[*c*]chromen-6-one dimethyl sulfoxide monosolvate), C_15_H_12_O_5_·C_2_H_6_OS, was isolated from an unidentified endophytic fungus (belonging to class Ascomycetes) of *Taxus sp*. In the crystal, both the alternariol 9-*O*-methyl ether (AME) and the dimethyl sulfoxide (DMSO) mol­ecules exhibit crystallographic mirror symmetry. One of the hy­droxy groups makes bifurcated hydrogen bonds, *viz.* an intra­molecular bond with the carbonyl group and an inter­molecular bond with the carbonyl group in an inversion-related AME mol­ecule. In the crystal, the AME mol­ecules are organized into stacks parallel with the *b* axis by π–π inter­actions between centrosymmetrically related mol­ecules [the distance between the centroid of the central ring and the centroid of the meth­oxy-substituted benzene ring in the next mol­ecule of the stack is 3.6184 (5) Å]. Pairs of DMSO mol­ecules, linked *via* centrosymmetric C—H⋯O contacts, are inserted into the voids created by the AME mol­ecules, making O—H⋯O and C—H⋯O contacts with the hosts.

## Related literature

For the bioactivity of AME and its precursor alternariol, see: Aly *et al.* (2008 ▶); Brugger *et al.* (2006 ▶); Pfeiffer *et al.* (2007 ▶); Miller *et al.* (2012 ▶). For their occurrence as contaminants in food and beverages, see: Lau *et al.* (2003 ▶). For the related crystal strucutre of alternariol, see: Dasari *et al.* (2012 ▶).
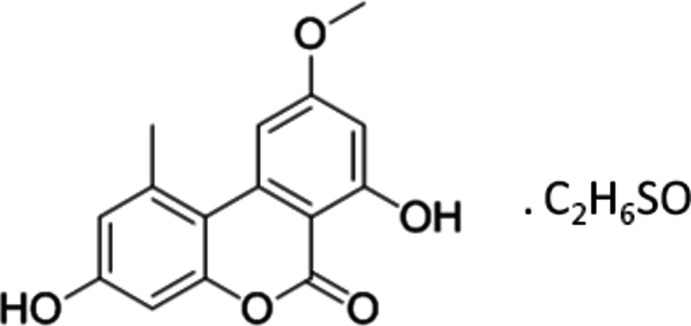


## Experimental

### 

#### Crystal data

C_15_H_12_O_5_·C_2_H_6_OS
*M* *_r_* = 350.37
Monoclinic, 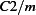

*a* = 18.8906 (8) Å
*b* = 6.8391 (3) Å
*c* = 15.3542 (8) Å
β = 126.815 (3)°
*V* = 1588.08 (13) Å^3^
*Z* = 4
Mo *K*α radiation
μ = 0.24 mm^−1^
*T* = 150 K
0.38 × 0.09 × 0.05 mm

#### Data collection

Bruker Kappa APEXII CCD diffractometer
Absorption correction: multi-scan (*SADABS*; Bruker, 2001 ▶) *T*_min_ = 0.916, *T*_max_ = 0.988
7177 measured reflections
1524 independent reflections
1382 reflections with *I* > 2σ(*I*)
*R*_int_ = 0.029

#### Refinement

*R*[*F* ^2^ > 2σ(*F* ^2^)] = 0.032
*wR*(*F* ^2^) = 0.093
*S* = 1.10
1524 reflections
144 parameters
3 restraints
H-atom parameters constrained
Δρ_max_ = 0.27 e Å^−3^
Δρ_min_ = −0.32 e Å^−3^

### 

Data collection: *APEX2* (Bruker, 2007 ▶); cell refinement: *SAINT* (Bruker, 2007 ▶); data reduction: *SAINT*; program(s) used to solve structure: *SHELXS97* (Sheldrick, 2008 ▶); program(s) used to refine structure: *SHELXL97* (Sheldrick, 2008 ▶); molecular graphics: *SHELXTL-Plus* (Sheldrick, 2008 ▶); software used to prepare material for publication: *SHELXTL-Plus*.

## Supplementary Material

Crystal structure: contains datablock(s) I, global. DOI: 10.1107/S1600536813012294/fy2088sup1.cif

Structure factors: contains datablock(s) I. DOI: 10.1107/S1600536813012294/fy2088Isup2.hkl

Supplementary material file. DOI: 10.1107/S1600536813012294/fy2088Isup3.cml

Additional supplementary materials:  crystallographic information; 3D view; checkCIF report

## Figures and Tables

**Table 1:** Hydrogen-bond geometry (Å, °)

*D*—H⋯*A*	*D*—H	H⋯*A*	*D*⋯*A*	*D*—H⋯*A*
O1—H1*O*1⋯O1*D* ^i^	0.80	1.82	2.617 (2)	175
O4—H1*O*4⋯O3	0.89	1.76	2.575 (2)	151
O4—H1*O*4⋯O3^ii^	0.89	2.59	3.162 (2)	123
C4—H4*C*⋯O1^iii^	0.93	2.62	3.467 (2)	152
C1*D*—H4⋯O4	0.96	2.70	3.401 (2)	130
C1*D*—H5⋯O2^ii^	0.96	2.66	3.2970 (19)	124

Symmetry codes: (i) 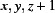; (ii) 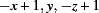; (iii) 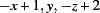.

**Fig. 1.**
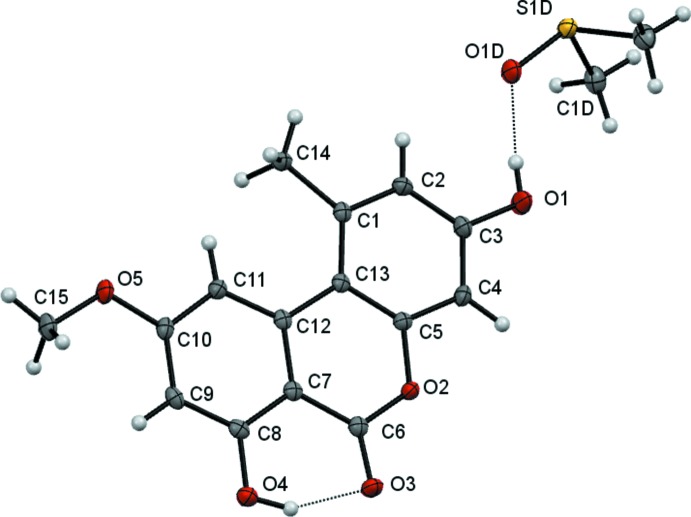
ORTEP view of the title compound showing the intramolecular and intermolecular AME···DMSO hydrogen bonds. Thermal ellipsoids are drawn at 40% probability level.

**Fig. 2.**
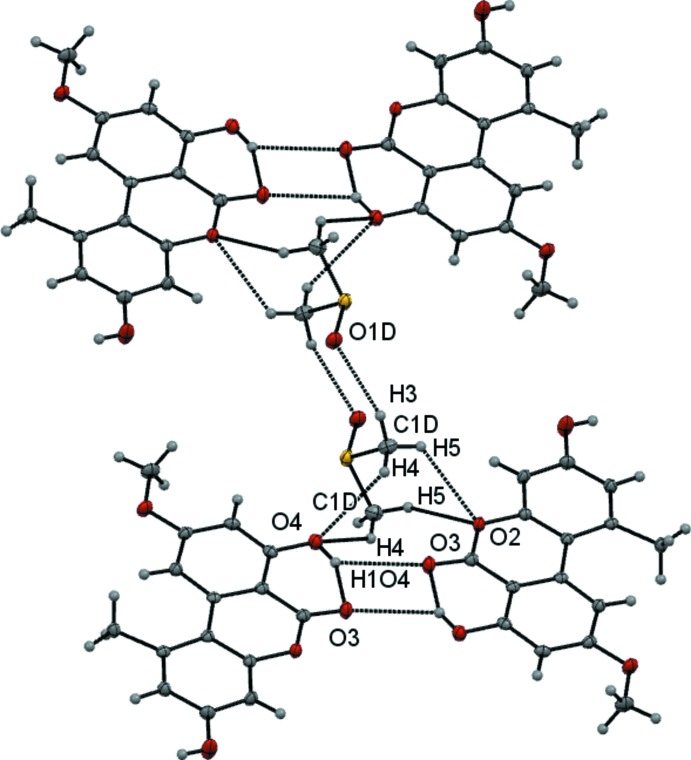
Molecular association between AME molecules and solvent DMSO molecules depicting a network of interactions.

**Fig. 3.**
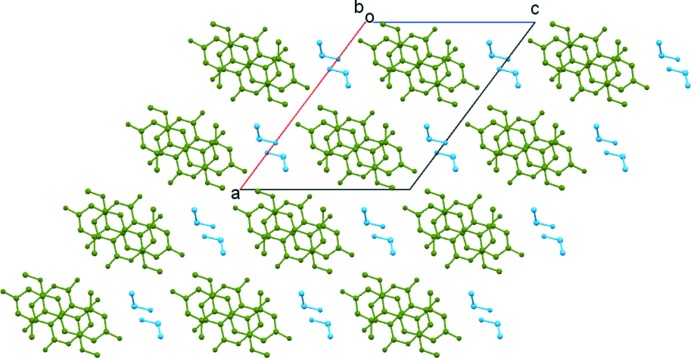
Packing of molecules viewed down the crystallographic b axis.

**Fig. 4.**
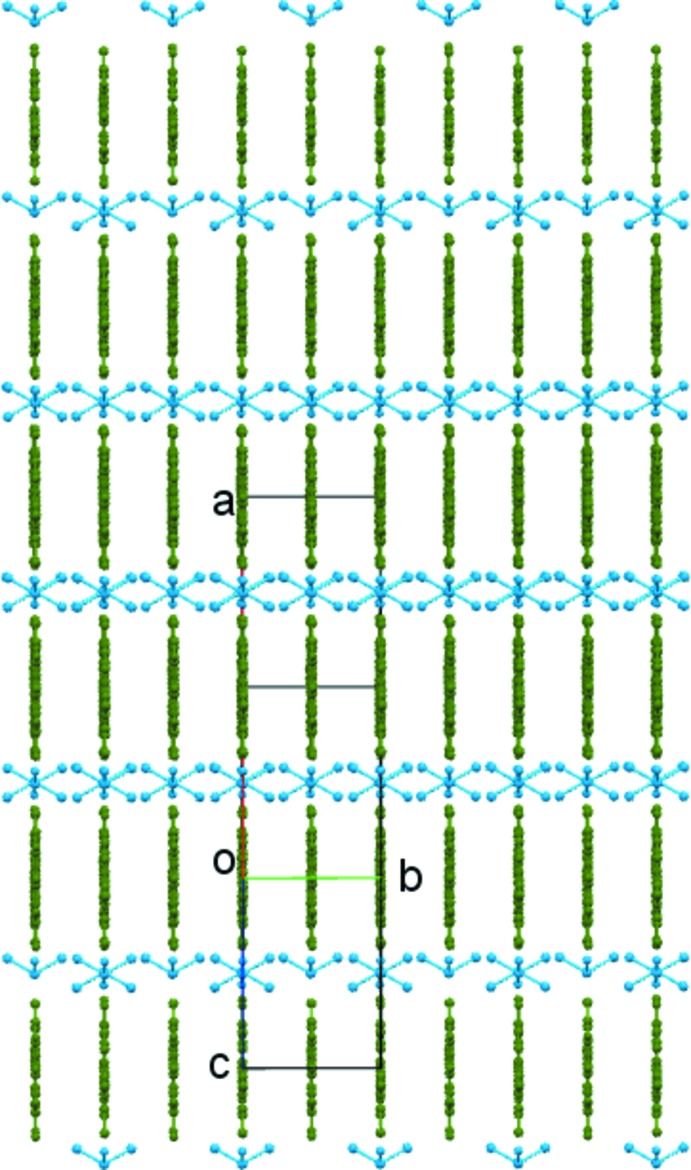
Packing of molecules along an arbitrary direction.

## supplementary crystallographic information

### Comment

Extracts of the fungal endophyte Ascomycete F53 have previously demonstrated promising anti-proliferative activity against multiple myeloma RPMI-8226 cells, and mild growth inhibitory activities against *Staphylococcus aureus*, *Escherichia coli* and *Cryptococcus albidus* (Miller *et al.*, 2012). The mycotoxin alternariol 9-*O*-methyl ether (AME) was previously thought to be produced soley by the fungal genus *Alternaria* however, its production has recently been reported from other fungal genera including *Phoma sp*.. Chemical profiling and genetic analysis of the fungus demonstrated the potential of the fungus to biosynthesize the mycotoxins, AOH, AME and other related derivatized secondary metabolites (data not shown).

Alternariol 9-*O*-methyl ether (AME; C_15_H_12_O_5_) and its precursor alternariol (AOH) are well known for their mammalian toxicity, mutagenic properties and mild antimicrobial properties.

Although AME has been well studied as a mycotoxin, the crystal structure was only recently reported by us (Dasari *et al.*, 2012). Due to the title compound's demonstrated varied biological activities it is a suitable candidate molecule to study its molecular arrangement in different crystalline environments. In this report, we present the DMSO solvated form of this compound.

An *ORTEP* view of the molecule and the solvent, DMSO, (Fig. 1) shows two O—H···O hydrogen bonds; one within the AME molecule (O4—H1O4···O3) and the other one between AME and DMSO (O1—H1O1···O1d). The molecular association involving significant interactions (Fig. 2) shows that the two DMSO molecules are held between two pairs of AME molecules making a network of C—H···O hydrogen bonds (Table 1). The two DMSO molecules are associated *via* centrosymmetric C1D—H3···O1D contacts. Each of these is attached through their methyl groups to two AME molecules *via* C—H···O contacts (Fig. 2 and Table 1). The two views of molecular packing looking down *b* axis (Fig. 3) and down an arbitrary direction (Fig. 4) show stacking of molecules with the DMSO molecules inserted into the crystal lattice without disturbing the parallel layer arrangement that was observed in the unsolvated form.

### Experimental

The fungal endophyte Ascomycete F53 was isolated from the Chinese medicinal plant *Taxus yunnanensis*, which was collected from mountainious area of Yunnan province in the Peoples Republic of China. A seed culture of Ascomycete F53 was used to innoculate 1*L* malt extract broth which was incubated for 21 days for the production of fungal secondary metabolites. The culture broth and mycelium were extracted with ethyl acetate (1*L*) to yield crude extract which was then fractionated on silica gel using a stepwise gradient of hexane to ethyl acetate and then with methanol to yield 12 fractions. The ethyl acetate fraction was further separated using C18 Sep-pak soild phase extraction column and eluted with a stepwise gradient of water to methanol. The fraction which eluted with 2:1 water/methanol yielded the title compound. The compound was dissolved in DMSO, and on slow evaporation of the solvent, formed plate like crystals.

### Refinement

All H-atoms (except for the two hydroxy H atoms) were positioned geometrically [C—H = 0.95 to 0.99 Å] and were refined using a riding-model approximation, with *U*_iso_(H) = 1.2 *U*_eq_(C) or 1.5 *U*_eq_(C-methyl). The torsional freedom of one of the methyl groups in the AME molecule was restricted by using *DFIX* restraints for intramolecular H···H distances [H14A···H11 and H14B···H11: 2.043 (1); H14C···H2: 2.190 (1)]. The hydroxyl oxygen peaks were located in the difference Fourier map and were refined in riding mode with their isotropic displacement parameters *U*_iso_(H) = 1.5 *U*_eq_(O).

### Crystal data

**Table:**

C_15_H_12_O_5_·C_2_H_6_OS	*F*(000) = 736
*M**_r_* = 350.37	*D*_x_ = 1.465 Mg m^−^^3^
Monoclinic, *C*2/*m*	Mo *K*α radiation, λ = 0.71073 Å
Hall symbol: -C 2y	Cell parameters from 4872 reflections
*a* = 18.8906 (8) Å	θ = 2.7–30.6°
*b* = 6.8391 (3) Å	µ = 0.24 mm^−^^1^
*c* = 15.3542 (8) Å	*T* = 150 K
β = 126.815 (3)°	Plate, colourless
*V* = 1588.08 (13) Å^3^	0.38 × 0.09 × 0.05 mm
*Z* = 4	

### Data collection

**Table:**

Bruker Kappa APEXII CCD diffractometer	1524 independent reflections
Radiation source: fine-focus sealed tube	1382 reflections with *I* > 2σ(*I*)
Graphite monochromator	*R*_int_ = 0.029
φ scans, and ω scans with κ offsets	θ_max_ = 25.0°, θ_min_ = 1.7°
Absorption correction: multi-scan (*SADABS*; Bruker, 2001)	*h* = −21→22
*T*_min_ = 0.916, *T*_max_ = 0.988	*k* = −8→8
7177 measured reflections	*l* = −18→18

### Refinement

**Table:**

Refinement on *F*^2^	Primary atom site location: structure-invariant direct methods
Least-squares matrix: full	Secondary atom site location: difference Fourier map
*R*[*F*^2^ > 2σ(*F*^2^)] = 0.032	Hydrogen site location: inferred from neighbouring sites
*wR*(*F*^2^) = 0.093	H-atom parameters constrained
*S* = 1.10	*w* = 1/[σ^2^(*F*_o_^2^) + (0.055*P*)^2^ + 0.9734*P*] where *P* = (*F*_o_^2^ + 2*F*_c_^2^)/3
1524 reflections	(Δ/σ)_max_ < 0.001
144 parameters	Δρ_max_ = 0.27 e Å^−^^3^
3 restraints	Δρ_min_ = −0.32 e Å^−^^3^

### Special details

**Table:**

Geometry. All e.s.d.'s (except the e.s.d. in the dihedral angle between two l.s. planes) are estimated using the full covariance matrix. The cell e.s.d.'s are taken into account individually in the estimation of e.s.d.'s in distances, angles and torsion angles; correlations between e.s.d.'s in cell parameters are only used when they are defined by crystal symmetry. An approximate (isotropic) treatment of cell e.s.d.'s is used for estimating e.s.d.'s involving l.s. planes.
Refinement. Refinement of *F*^2^ against ALL reflections. The weighted *R*-factor *wR* and goodness of fit *S* are based on *F*^2^, conventional *R*-factors *R* are based on *F*, with *F* set to zero for negative *F*^2^. The threshold expression of *F*^2^ > σ(*F*^2^) is used only for calculating *R*-factors(gt) *etc*. and is not relevant to the choice of reflections for refinement. *R*-factors based on *F*^2^ are statistically about twice as large as those based on *F*, and *R*- factors based on ALL data will be even larger.

### Fractional atomic coordinates and isotropic or equivalent isotropic displacement parameters (Å^2^)

**Table:**

	*x*	*y*	*z*	*U*_iso_*/*U*_eq_	Occ. (<1)
O1	0.39016 (10)	0.0000	0.94667 (11)	0.0391 (4)	
H1O1	0.3537	0.0000	0.9575	0.059*	
O2	0.42281 (8)	0.0000	0.67046 (11)	0.0206 (3)	
O3	0.45897 (9)	0.0000	0.55957 (11)	0.0265 (4)	
O4	0.34069 (9)	0.0000	0.35176 (11)	0.0237 (3)	
H1O4	0.3931	0.0000	0.4162	0.036*	
O5	0.04062 (9)	0.0000	0.22240 (11)	0.0268 (4)	
C1	0.21796 (12)	0.0000	0.64985 (15)	0.0174 (4)	
C2	0.25831 (11)	0.0000	0.76077 (13)	0.0205 (4)	
H2	0.2229	0.0000	0.7842	0.025*	
C3	0.34992 (13)	0.0000	0.83847 (15)	0.0227 (5)	
C4	0.40259 (13)	0.0000	0.80403 (15)	0.0209 (4)	
H4C	0.4639	0.0000	0.8540	0.025*	
C5	0.36216 (13)	0.0000	0.69341 (16)	0.0173 (4)	
C6	0.39827 (13)	0.0000	0.56791 (16)	0.0190 (4)	
C7	0.30546 (12)	0.0000	0.47951 (15)	0.0172 (4)	
C8	0.28052 (13)	0.0000	0.37232 (16)	0.0185 (4)	
C9	0.19222 (13)	0.0000	0.28315 (15)	0.0202 (4)	
H9	0.1757	0.0000	0.2126	0.024*	
C10	0.12935 (13)	0.0000	0.30239 (16)	0.0192 (4)	
C11	0.15201 (12)	0.0000	0.40773 (13)	0.0194 (4)	
H11	0.1077	0.0000	0.4170	0.023*	
C12	0.24039 (13)	0.0000	0.49846 (16)	0.0161 (4)	
C13	0.27066 (12)	0.0000	0.61190 (15)	0.0162 (4)	
C14	0.11825 (10)	0.0000	0.57677 (11)	0.0218 (4)	
H14A	0.0970	−0.1146	0.5317	0.033*	0.50
H14B	0.0970	0.1146	0.5317	0.033*	0.50
H14C	0.0974	0.0000	0.6204	0.033*	
C15	0.01044 (14)	0.0000	0.11184 (16)	0.0292 (5)	
H15A	−0.0530	0.0000	0.0641	0.044*	
H15B	0.0320	−0.1146	0.0986	0.044*	0.50
H15C	0.0320	0.1146	0.0986	0.044*	0.50
S1D	0.30260 (3)	0.0000	0.10913 (4)	0.02538 (19)	
O1D	0.27939 (9)	0.0000	−0.00407 (11)	0.0298 (4)	
C1D	0.37818 (10)	0.1967 (2)	0.17933 (12)	0.0285 (4)	
H4	0.3992	0.1972	0.2538	0.043*	
H3	0.3489	0.3181	0.1455	0.043*	
H5	0.4273	0.1808	0.1765	0.043*	

### Atomic displacement parameters (Å^2^)

**Table:**

	*U*^11^	*U*^22^	*U*^33^	*U*^12^	*U*^13^	*U*^23^
O1	0.0209 (8)	0.0812 (13)	0.0132 (7)	0.000	0.0091 (7)	0.000
O2	0.0136 (7)	0.0319 (7)	0.0157 (7)	0.000	0.0084 (6)	0.000
O3	0.0170 (7)	0.0435 (9)	0.0217 (8)	0.000	0.0131 (6)	0.000
O4	0.0209 (7)	0.0340 (8)	0.0190 (7)	0.000	0.0134 (6)	0.000
O5	0.0173 (7)	0.0422 (9)	0.0130 (7)	0.000	0.0048 (6)	0.000
C1	0.0179 (10)	0.0161 (9)	0.0162 (9)	0.000	0.0092 (8)	0.000
C2	0.0195 (10)	0.0265 (10)	0.0188 (10)	0.000	0.0132 (9)	0.000
C3	0.0221 (10)	0.0303 (11)	0.0142 (10)	0.000	0.0100 (9)	0.000
C4	0.0135 (9)	0.0284 (10)	0.0151 (10)	0.000	0.0054 (8)	0.000
C5	0.0166 (9)	0.0180 (9)	0.0180 (10)	0.000	0.0108 (8)	0.000
C6	0.0189 (10)	0.0190 (9)	0.0184 (10)	0.000	0.0108 (8)	0.000
C7	0.0181 (10)	0.0152 (9)	0.0176 (10)	0.000	0.0103 (9)	0.000
C8	0.0236 (10)	0.0152 (9)	0.0200 (10)	0.000	0.0150 (9)	0.000
C9	0.0257 (11)	0.0198 (9)	0.0140 (9)	0.000	0.0113 (9)	0.000
C10	0.0165 (9)	0.0190 (9)	0.0157 (10)	0.000	0.0062 (8)	0.000
C11	0.0164 (10)	0.0226 (9)	0.0176 (10)	0.000	0.0094 (9)	0.000
C12	0.0188 (9)	0.0118 (8)	0.0161 (9)	0.000	0.0097 (8)	0.000
C13	0.0163 (9)	0.0145 (9)	0.0165 (10)	0.000	0.0092 (8)	0.000
C14	0.0174 (10)	0.0307 (10)	0.0190 (10)	0.000	0.0119 (9)	0.000
C15	0.0266 (12)	0.0389 (12)	0.0132 (10)	0.000	0.0072 (9)	0.000
S1D	0.0166 (3)	0.0424 (3)	0.0167 (3)	0.000	0.0097 (2)	0.000
O1D	0.0221 (8)	0.0481 (9)	0.0152 (7)	0.000	0.0090 (6)	0.000
C1D	0.0306 (8)	0.0267 (8)	0.0234 (8)	0.0054 (6)	0.0136 (7)	0.0019 (6)

### Geometric parameters (Å, º)

**Table:**

O1—C3	1.351 (2)	C7—C12	1.423 (3)
O1—H1O1	0.8003	C8—C9	1.386 (3)
O2—C6	1.349 (2)	C9—C10	1.385 (3)
O2—C5	1.386 (2)	C9—H9	0.9300
O3—C6	1.228 (2)	C10—C11	1.403 (2)
O4—C8	1.350 (2)	C11—C12	1.393 (3)
O4—H1O4	0.8859	C11—H11	0.9300
O5—C10	1.360 (2)	C12—C13	1.472 (3)
O5—C15	1.431 (2)	C14—H14A	0.9600
C1—C2	1.388 (2)	C14—H14B	0.9600
C1—C13	1.423 (3)	C14—H14C	0.9600
C1—C14	1.508 (2)	C15—H15A	0.9600
C2—C3	1.394 (3)	C15—H15B	0.9600
C2—H2	0.9300	C15—H15C	0.9600
C3—C4	1.378 (3)	S1D—O1D	1.5166 (15)
C4—C5	1.383 (3)	S1D—C1D	1.7790 (15)
C4—H4C	0.9300	S1D—C1D^i^	1.7790 (15)
C5—C13	1.401 (3)	C1D—H4	0.9600
C6—C7	1.437 (3)	C1D—H3	0.9600
C7—C8	1.415 (3)	C1D—H5	0.9600
			
C3—O1—H1O1	109.6	O5—C10—C11	113.60 (17)
C6—O2—C5	122.59 (15)	C9—C10—C11	122.51 (17)
C8—O4—H1O4	105.8	C12—C11—C10	120.51 (16)
C10—O5—C15	118.07 (16)	C12—C11—H11	119.7
C2—C1—C13	119.87 (17)	C10—C11—H11	119.7
C2—C1—C14	115.84 (16)	C11—C12—C7	117.39 (17)
C13—C1—C14	124.30 (16)	C11—C12—C13	124.49 (17)
C1—C2—C3	122.50 (18)	C7—C12—C13	118.13 (17)
C1—C2—H2	118.7	C5—C13—C1	115.19 (17)
C3—C2—H2	118.7	C5—C13—C12	116.97 (17)
O1—C3—C4	117.92 (18)	C1—C13—C12	127.83 (17)
O1—C3—C2	123.20 (18)	C1—C14—H14A	109.5
C4—C3—C2	118.88 (18)	C1—C14—H14B	109.5
C3—C4—C5	118.47 (18)	H14A—C14—H14B	109.5
C3—C4—H4C	120.8	C1—C14—H14C	109.5
C5—C4—H4C	120.8	H14A—C14—H14C	109.5
C4—C5—O2	112.33 (16)	H14B—C14—H14C	109.5
C4—C5—C13	125.09 (18)	O5—C15—H15A	109.5
O2—C5—C13	122.58 (17)	O5—C15—H15B	109.5
O3—C6—O2	115.63 (17)	H15A—C15—H15B	109.5
O3—C6—C7	126.07 (18)	O5—C15—H15C	109.5
O2—C6—C7	118.30 (17)	H15A—C15—H15C	109.5
C8—C7—C12	120.78 (17)	H15B—C15—H15C	109.5
C8—C7—C6	117.79 (17)	O1D—S1D—C1D	105.68 (6)
C12—C7—C6	121.43 (18)	O1D—S1D—C1D^i^	105.68 (6)
O4—C8—C9	116.93 (17)	C1D—S1D—C1D^i^	98.23 (10)
O4—C8—C7	122.14 (17)	S1D—C1D—H4	109.5
C9—C8—C7	120.93 (18)	S1D—C1D—H3	109.5
C10—C9—C8	117.89 (18)	H4—C1D—H3	109.5
C10—C9—H9	121.1	S1D—C1D—H5	109.5
C8—C9—H9	121.1	H4—C1D—H5	109.5
O5—C10—C9	123.89 (18)	H3—C1D—H5	109.5
			
C13—C1—C2—C3	0.000 (1)	C15—O5—C10—C11	180.0
C14—C1—C2—C3	180.0	C8—C9—C10—O5	180.0
C1—C2—C3—O1	180.0	C8—C9—C10—C11	0.0
C1—C2—C3—C4	0.000 (1)	O5—C10—C11—C12	180.0
O1—C3—C4—C5	180.000 (1)	C9—C10—C11—C12	0.0
C2—C3—C4—C5	0.000 (1)	C10—C11—C12—C7	0.0
C3—C4—C5—O2	180.0	C10—C11—C12—C13	180.0
C3—C4—C5—C13	0.0	C8—C7—C12—C11	0.0
C6—O2—C5—C4	180.0	C6—C7—C12—C11	180.0
C6—O2—C5—C13	0.0	C8—C7—C12—C13	180.0
C5—O2—C6—O3	180.0	C6—C7—C12—C13	0.0
C5—O2—C6—C7	0.0	C4—C5—C13—C1	0.0
O3—C6—C7—C8	0.0	O2—C5—C13—C1	180.0
O2—C6—C7—C8	180.0	C4—C5—C13—C12	180.0
O3—C6—C7—C12	180.0	O2—C5—C13—C12	0.000 (1)
O2—C6—C7—C12	0.0	C2—C1—C13—C5	0.000 (1)
C12—C7—C8—O4	180.0	C14—C1—C13—C5	180.0
C6—C7—C8—O4	0.0	C2—C1—C13—C12	180.0
C12—C7—C8—C9	0.0	C14—C1—C13—C12	0.0
C6—C7—C8—C9	180.0	C11—C12—C13—C5	180.0
O4—C8—C9—C10	180.0	C7—C12—C13—C5	0.0
C7—C8—C9—C10	0.0	C11—C12—C13—C1	0.0
C15—O5—C10—C9	0.0	C7—C12—C13—C1	180.0

Symmetry code: (i) *x*, −*y*, *z*.

### Hydrogen-bond geometry (Å, º)

**Table:**

*D*—H···*A*	*D*—H	H···*A*	*D*···*A*	*D*—H···*A*
O1—H1*O*1···O1*D*^ii^	0.80	1.82	2.617 (2)	175
O4—H1*O*4···O3	0.89	1.76	2.575 (2)	151
O4—H1*O*4···O3^iii^	0.89	2.59	3.162 (2)	123
C4—H4*C*···O1^iv^	0.93	2.62	3.467 (2)	152
C1*D*—H4···O4	0.96	2.70	3.401 (2)	130
C1*D*—H5···O2^iii^	0.96	2.66	3.2970 (19)	124

Symmetry codes: (ii) *x*, *y*, *z*+1; (iii) −*x*+1, *y*, −*z*+1; (iv) −*x*+1, *y*, −*z*+2.
